# Multielectrode Teflon electrochemical nanocatalyst investigation system

**DOI:** 10.1016/j.mex.2015.04.004

**Published:** 2015-04-22

**Authors:** Nejc Hodnik

**Affiliations:** a Max-Planck-Institut für Eisenforschung GmbH, Max-Planck Str. 1, 40237 Düsseldorf, Germany; b National Institute of Chemistry, Hajdrihova 19, 1000 Ljubljana, Slovenia

**Keywords:** Multielectrode Teflon electrochemical nanocatalyst investigation system, Electrocatalysis, Electrochemical cell, Multielectrode electrochemical cell, Oxygen reduction reaction, Platinum activity

## Abstract

The most common approach in the search for the optimal low temperature fuel cell catalyst remains “trial and error”. Therefore, large numbers of different potential catalytic materials need to be screened. The well-established and most commonly used method for testing catalytic electrochemical activity under well-defined hydrodynamics is still thin film rotating disc electrode (TF-RDE). Typically this method is very time consuming and is subjected to impurity problems. In order to avoid these issues a new multielectrode electrochemical cell design is presented, where 8 different electrocatalysts can be measured simultaneously at identical conditions.

The major advantages over TF-RDE method are:

• Faster catalyst screening times.
• Greater impurity tolerance.
• The option of internal standard.

## Graphical abstract

**Figure:**
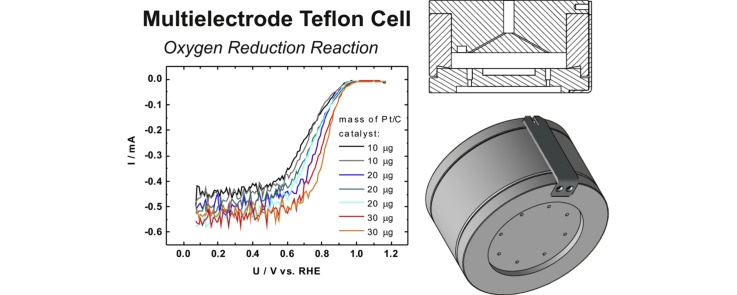

## Method details

### Background information

There are two methods commonly used to investigate low temperature fuel cell catalyst activity. First one is membrane electrode assembly (MEA) method which is actually an essential part of a real proton exchange membrane fuel cell (PEM-FC). It is a sandwich (composite) of a proton exchange membrane (nafion), catalyst layer and a gas diffusion layer. The main practical problems associated with this method are high costs, large consumption of electrocatalyst, excessive waste of time due to assembling of the MEA and complex interpretation of results due to the large number of parameters that influence a fuel cell performance (water management, membrane thickness, catalyst porosity, etc.). This makes MEA method very unpractical for fast screening of new materials. With the thin film rotating disc electrode (TF-RDE) the number of parameters reduces significantly. The catalyst is deposited on a glassy carbon electrode in a form of a thin film and then measured with classical 3 electrode electrochemical setup under defined mass transport conditions [1], [2]. In addition to much easier and less complex interpretation compared to MEA it also consumes significantly less time and catalyst [3]. RDE has been proven to produce results that are in accordance with MEA activity measurements. This method is used in almost every laboratory testing electrocatalysts. Frequently studied reactions are oxygen reduction, methanol or ethanol oxidation, hydrogen oxidation, oxygen evolution, nitrous oxide reduction, and more [4]. Still, this method has some potential problems, which are related mainly to the impurities that can alter the real kinetic of reactions like ORR [5]. This is especially important in alkaline electrolyte [6]. In addition, typical electrochemical characterization experiment of a single sample takes approximately 2–3 h, which is also suitable for fast screening of electrocatalysts. One alternative is to use carbon microfiber as a working electrode (WE) [7]. However in that case it is difficult to control the amount of a deposited catalyst, therefore, only specific activity (without mass activity) can be obtained.

In this study we propose a new design of an electrochemical cell that enables 8 electrochemical evaluations at the same time. Compared to the multi potentiostat systems that have multiple counter (CE) and reference electrodes (RE), commonly used in battery research, our approach is to utilise only one reference and one counter electrode. Therefore we can use only one potentiostat with addition of special module enabling simultaneous control over potentials of 8 working electrodes. This is another important factor that brings down the costs. Control over mass transport of reactive species to the electrode surface (hydrodynamics) is achieved with magnetic stirrer. This is a clear advantage over similar commercial systems [8].

### Advantages of multielectrode Teflon cell over TF-RDE glass cell

– Roughly 8 times faster sample characterisation.
– Identical iR drop for all WE; same distance between WE and CE and RE for all 8 electrodes.
– Identical conditions for all samples; same electrolyte, temperature, impurities and reactant concentration.
– The option of internal benchmark standard with known activity parameters provides the information and control over the measurements conditions like pH, impurities, temperature, reactant concentration, etc.
– Ability to measure in alkaline electrolytes [6].

### Disadvantages of multielectrode Teflon cell over TF RDE

- No as well defined hydrodynamics, therefore, lower control over the mass flux of reactants. Optimisation of magnetic stirring is needed in the future.
- Activities are not directly comparable to TF-RDE due to different mass transport [9].

## Multielectrode Teflon cell technical description

A new electrochemical cell has been constructed for the purpose of utilising eight working electrodes with one counter and one reference electrode with one potentiostat. In addition to potentiostat (CompactStat.e) special module (MultiWE32) is needed to utilise multiple working electrodes. This can also be achieved with homemade circuit design module consisting of several trans-resistance amplifiers in parallel, one for each WE [10]. This enables simultaneous biasing of 8 working electrodes with just one reference and one counter electrode. Further, specific details about this principle can be found in PhD thesis of Dr. Gustav Karl Henrik Wiberg [10]. We want to note that one could also use an electrochemical multiplexer or just a potentiostat and measure each WE separately. However, this would be more time consuming.

The distances from the working electrodes to the reference and also to the counter electrode are all exactly the same, thus, all WE exhibit the same iR drop [11]. The defined flux of the reactant and products to or from the surface through laminar film is achieved by mixing the solution with a magnetic stick controlled by external magnetic stirrer (placed underneath the cell). In TF-RDE the same is achieved by rotating the electrode. There is no interruption of measurement signal by the magnetic stirrer. However, further optimization of the stirring is still possible. All parts of the cell are made of Teflon enabling the use of alkaline electrolyte, which is not appropriate for the glass cells [6].

**Fig. 1:**
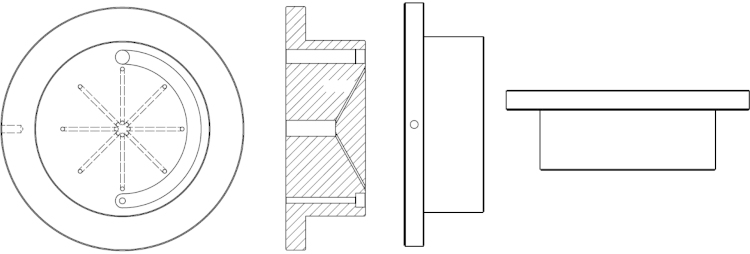
It consists of 8 channels leading from a reference electrode channel in the middle to 8 exits positioned above each WE. Additionally channels for gas inlet and outlet are drilled.

**Fig. 2:**
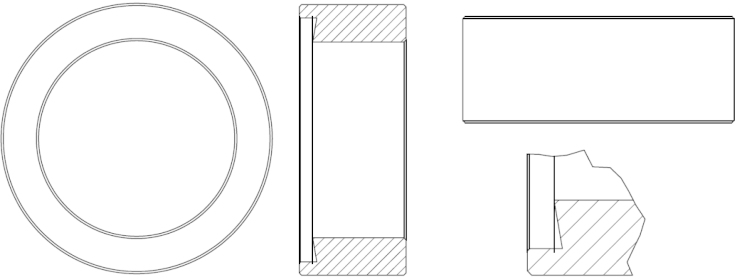
It is cylindrical shape and presents the wall of electrochemical cell. It is screwed to the bottom part. In order to avoid any leaking tight contact is made to bottom part with a shaped corner edge.

**Fig. 3:**
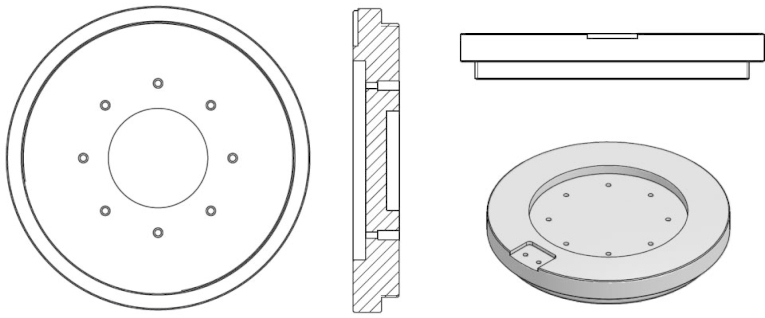
It consists of Teflon disc with concentrically positioned 8 glassy carbon discs (with diameter of 2 mm) as working electrodes. From the bottom side each electrode was electrically contacted with a golden pin and a wire (Fig. 5; also one wire was added for grounding all of the cables). In the middle of a Teflon disc a circular hole was drilled for the magnetic stirring stick. Counter electrode (platinum wire) is placed concentrically around working electrodes near the edge of the bottom part (see first image in Fig. 5).

The Teflon cell consists of three parts:

1. Upper Teflon part or the cover (Fig. 1).
2. Middle part or the wall (Fig. 2).
3. Bottom part or the electrodes (Fig. 3).

Further details about dimensions of the cell design can be found in supplementary information.

### Example of measurement protocol

The performance of the method is exemplified here on a Pt based carbon supported low temperature fuel cell catalyst (for more information about catalysts look in Refs. [4], [12], [13]). However, we must note that also any other powder-type catalyst can be used.

**Fig. 4:**
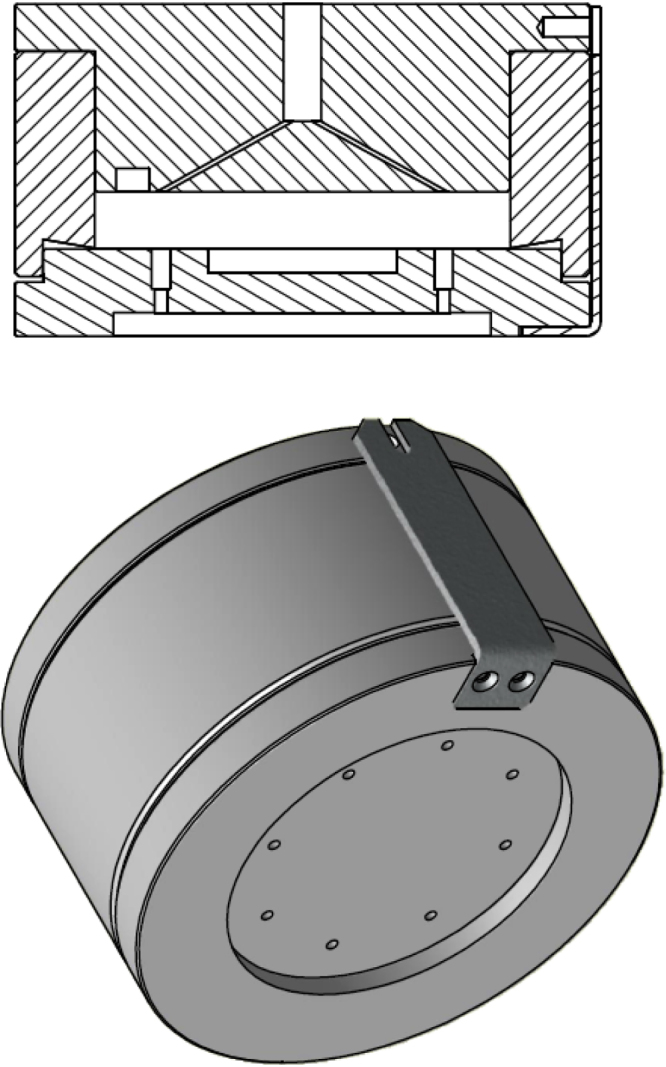
Multielectrode Teflon cell assembly.

**Fig. 5:**
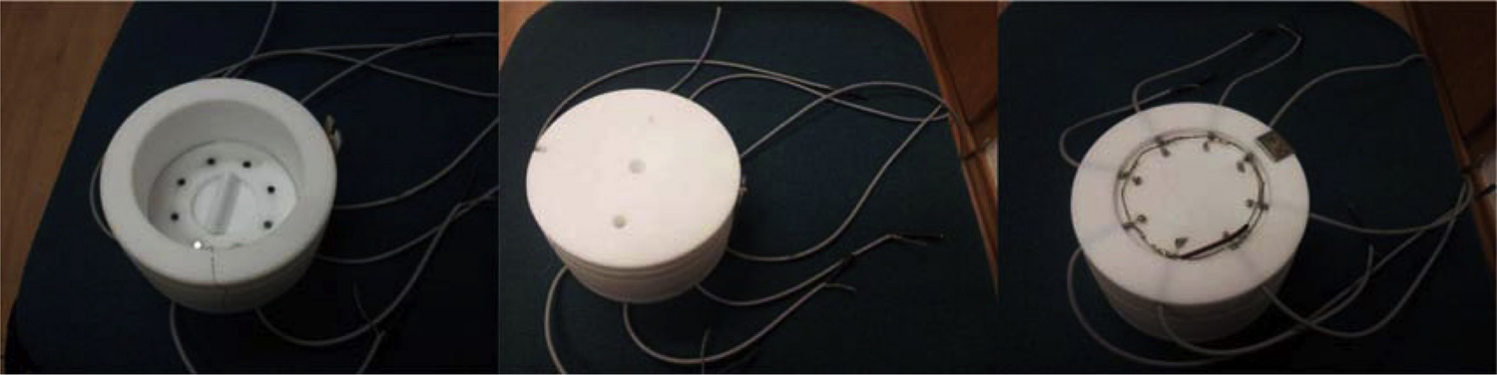
The multielectrode Teflon cell assembly pictures.

**Fig. 6:**
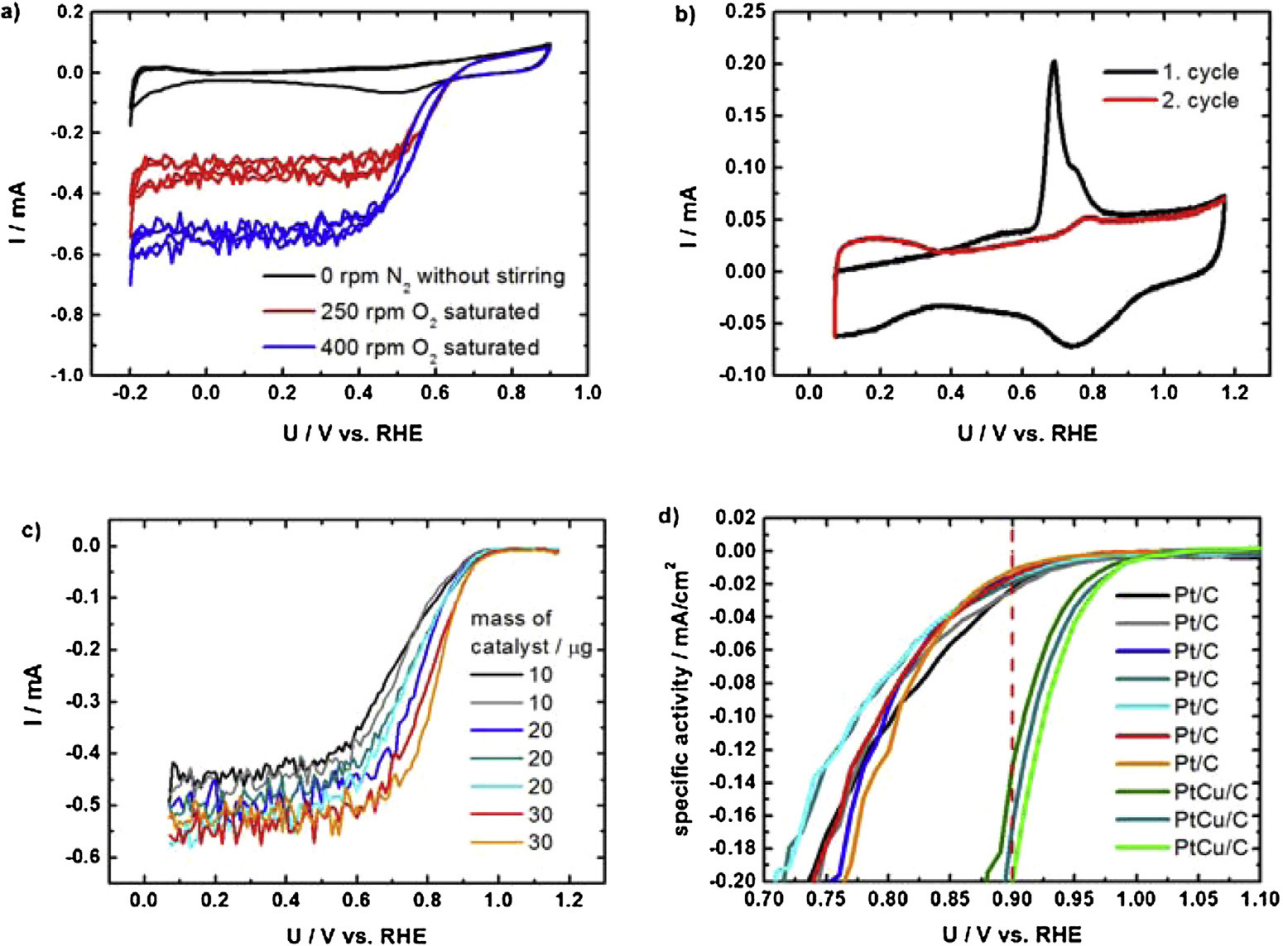
(a) baseline and ORR polarization curves at two different rotations, (b) CO stripping experiment, (d) ORR polarization curves with subtracted background capacitive currents with different catalyst loadings and (c) Pt/C and PtCu/C (loading was 20 μg).

**Fig. 7:**
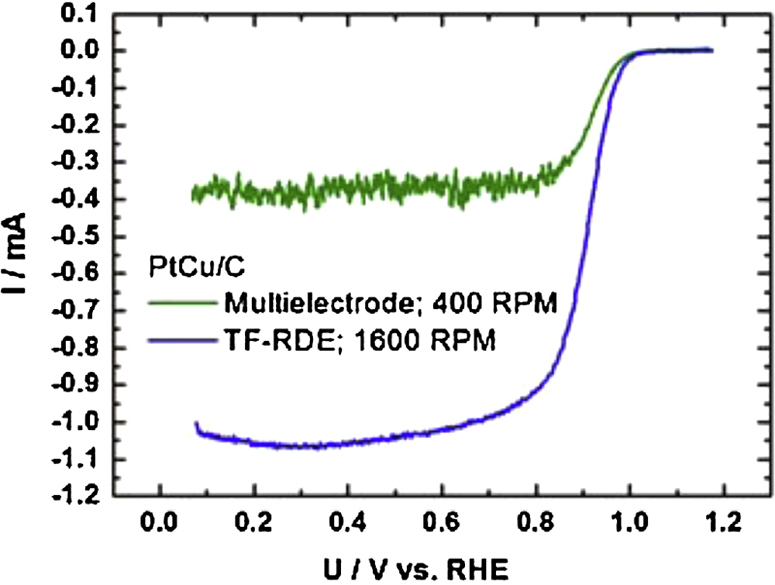
Comparison of ORR polarization curves measured with multielectrode Teflon and TF-RDE cell of PtCu/C with the same catalyst loading.

1. Catalyst thin film preparation: 5 mg of catalyst water dispersed in 5 ml Milli-Q water in water bath-type ultrasonicator for 1 h. After homogeneous and stable suspension was obtained 10–30 μl was drop casted over each glassy carbon electrode. To prevent detachment 5 μl of Nafion (5 wt%, Fluka) suspension in isopropyl alcohol (pro analysis, Merck) in 1/50 ratio was drop casted over the dried films. The procedure is similar as in Ref. [12].
2. Multielectrode electrochemical cell assembly: As can be seen in Fig. 4, Fig. 5 the electrochemical cell was assembled by screwing the lower Teflon part with glassy carbon electrodes together with the middle oval Teflon part. Then the platinum wire as counter electrode was placed inside (see Fig. 5). Afterwards the electrolyte is filled and enclosed by the upper part. Reference electrode is inserted in the reference compartment, where it should be in contact with electrolyte. Finally, electrodes are connected to the potentiostat and gas connections are made. The channels leading to the reference electrode in the upper part should be centred above the working electrodes in order to lower the iR drop.
3. Electrochemical measurement: Here the example of Pt based low temperature fuel cell catalyst measurement protocol is given: first initiation or activation cycles are performed in order to obtain stable cyclic voltammogram. This is done in a saturated electrolyte with inert gas (Ar or N_2_) that was purged for at least 20 min (with magnetic stirrer turned on). Then the activity is measured in oxygen saturated solution (Fig. 6a). Lastly, electrochemical surface area is obtained with an integration of CO stripping curve (Fig. 6b). The procedure is similar as in the TF-RDE experiment [13]. In Fig. 6c we can see a typical multielectrode experiment where different loadings of Pt/C standard were measured at the same time. In Fig. 6d we can see onsets of two different electrocatalyst. It is clear that PtCu/C exhibits higher ORR activity then Pt/C (more about the nature of catalyst and their activates can be found in Ref. [4]). Compared to TF-RDE experiment performed with PtCu/C one can observe the same onset potential, which indicates the same catalyst activity. However, there is a clear difference in the diffusion region due to different hydrodynamics (Fig. 7). If interested in methanol oxidation activity CO stripping is measured before adding methanol. Optionally one could also perform other electrochemical measurements such as different reactions (hydrogen, oxidation, ethanol oxidation, nitrous oxide reduction, oxygen evolution, etc.), degradation protocols (cycling or potential hold) [12], treatment of either identical location TEM grids or SEM graphite holders [13].
4. Cleaning of the multielectrode Teflon cell: Cell is disassembled and dipped in to the Piranha solution overnight. Working glassy carbon electrodes are polished all at the same time using alumina slurry in a micro-cloth before every experiment. Thorough rinsing with Milli-Q water is necessary before use.
