# Supplementary figures and images for: Multielectrode Teflon electrochemical nanocatalyst investigation system
Source: MethodsX. 2015 Apr 22;2:204–10. doi: 10.1016/j.mex.2015.04.004 (PMC4487726; doi:10.1016/j.mex.2015.04.004)

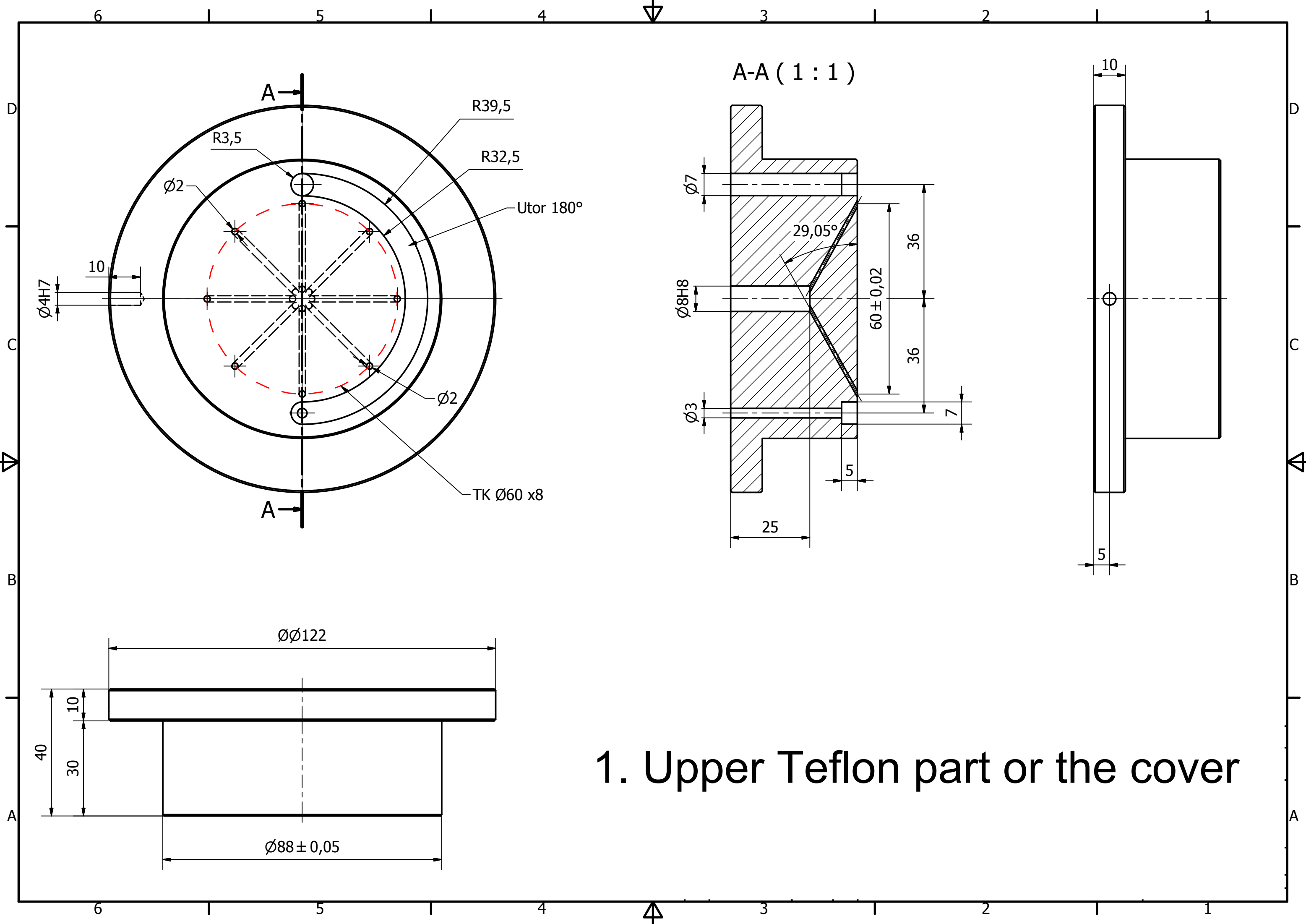

1. Upper Teflon part or the cover

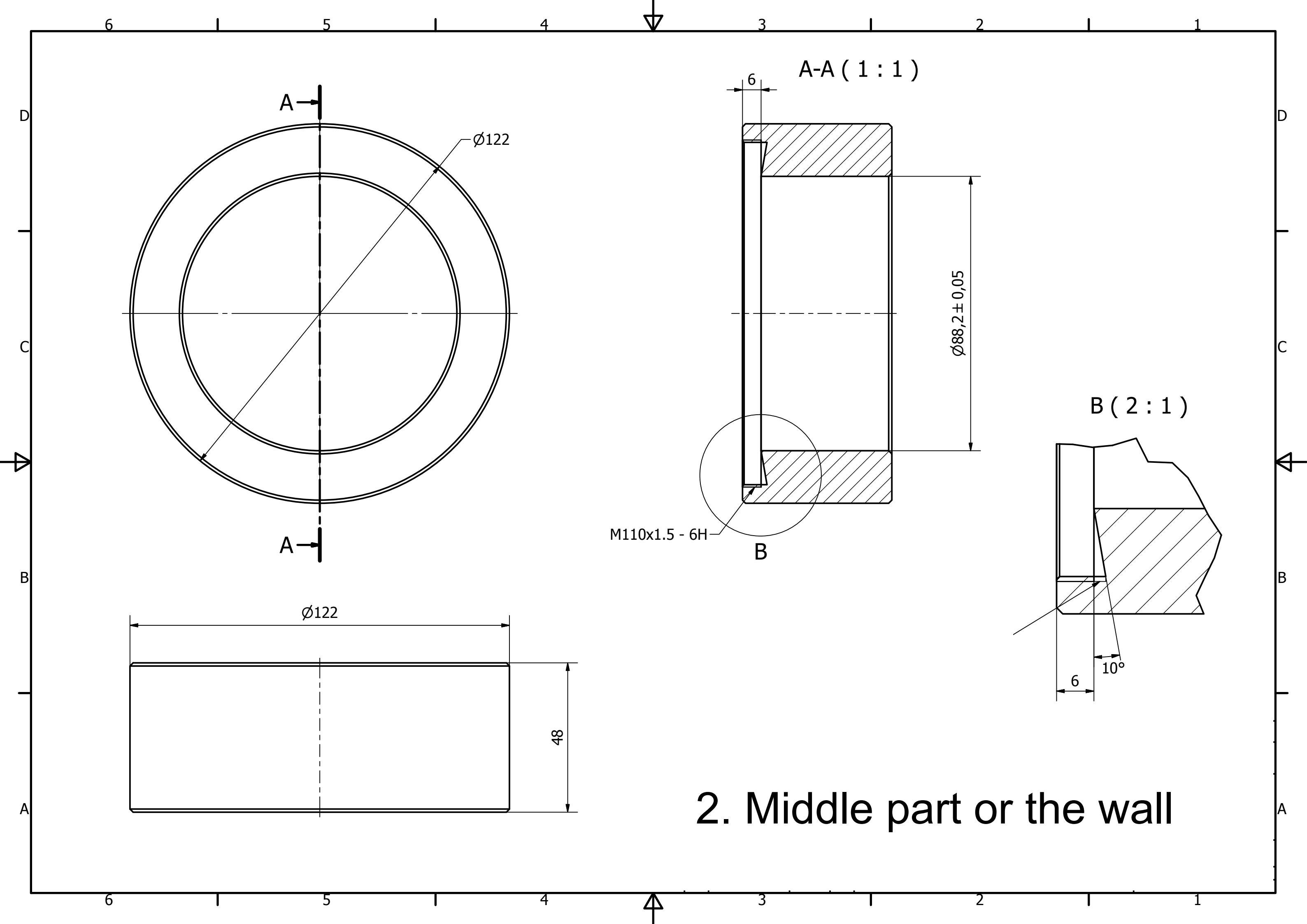

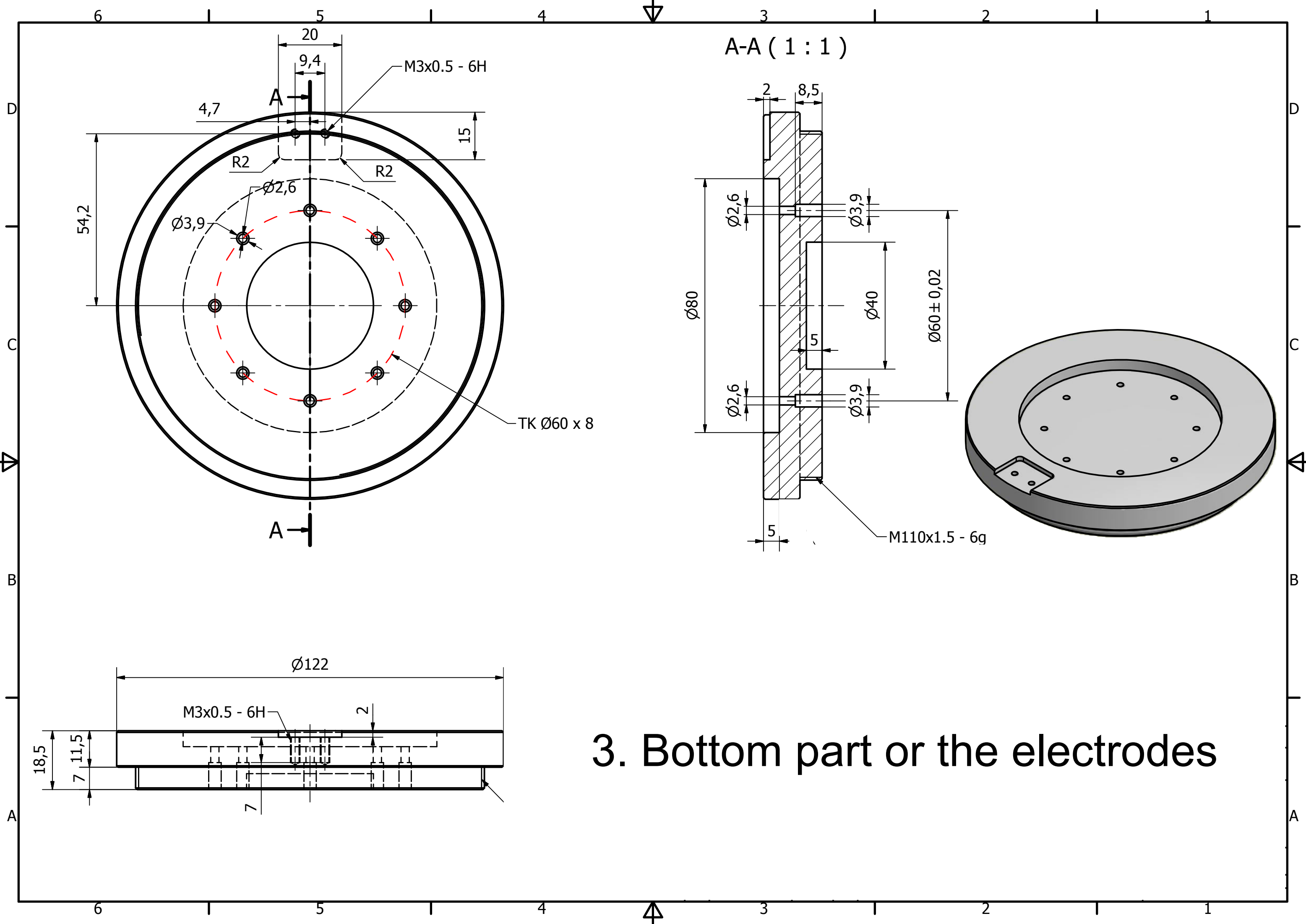

3. Bottom part or the electrodes

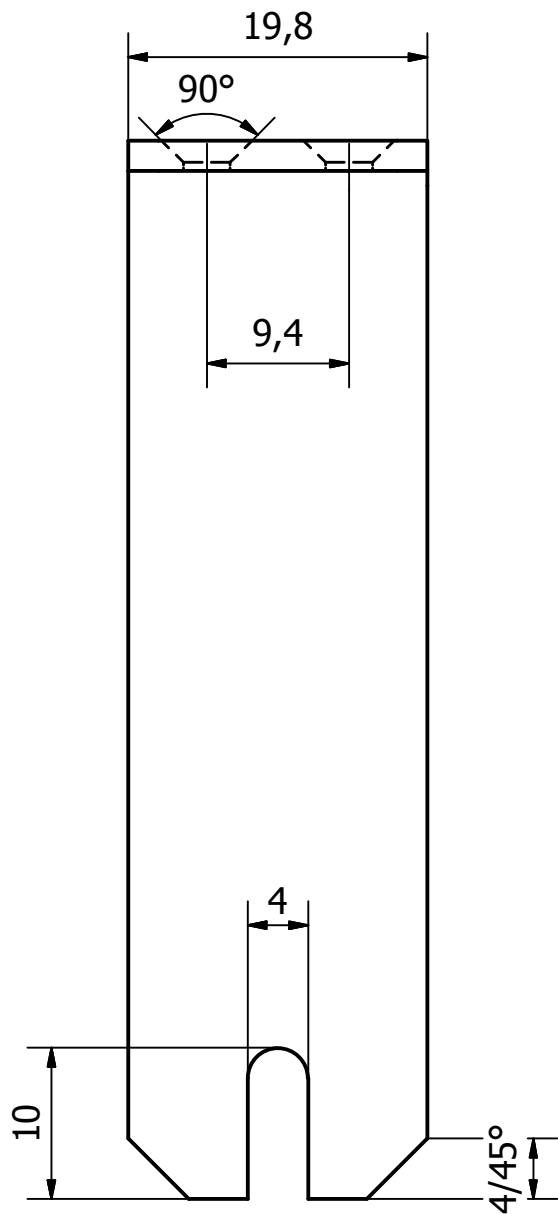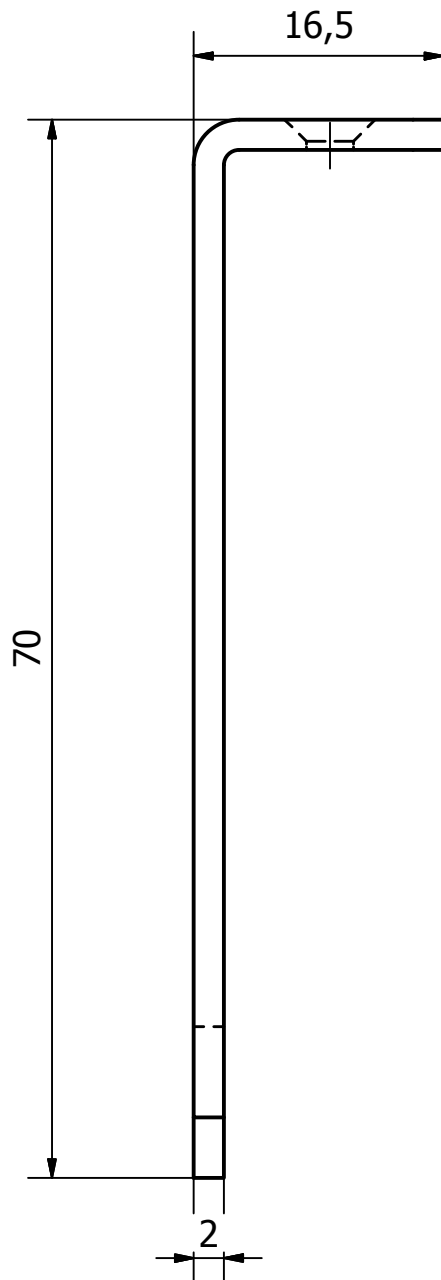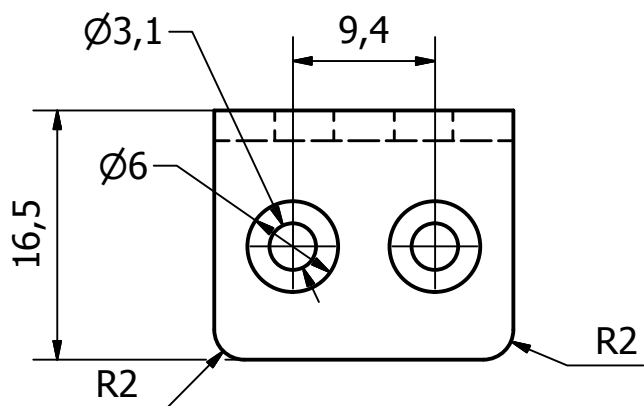

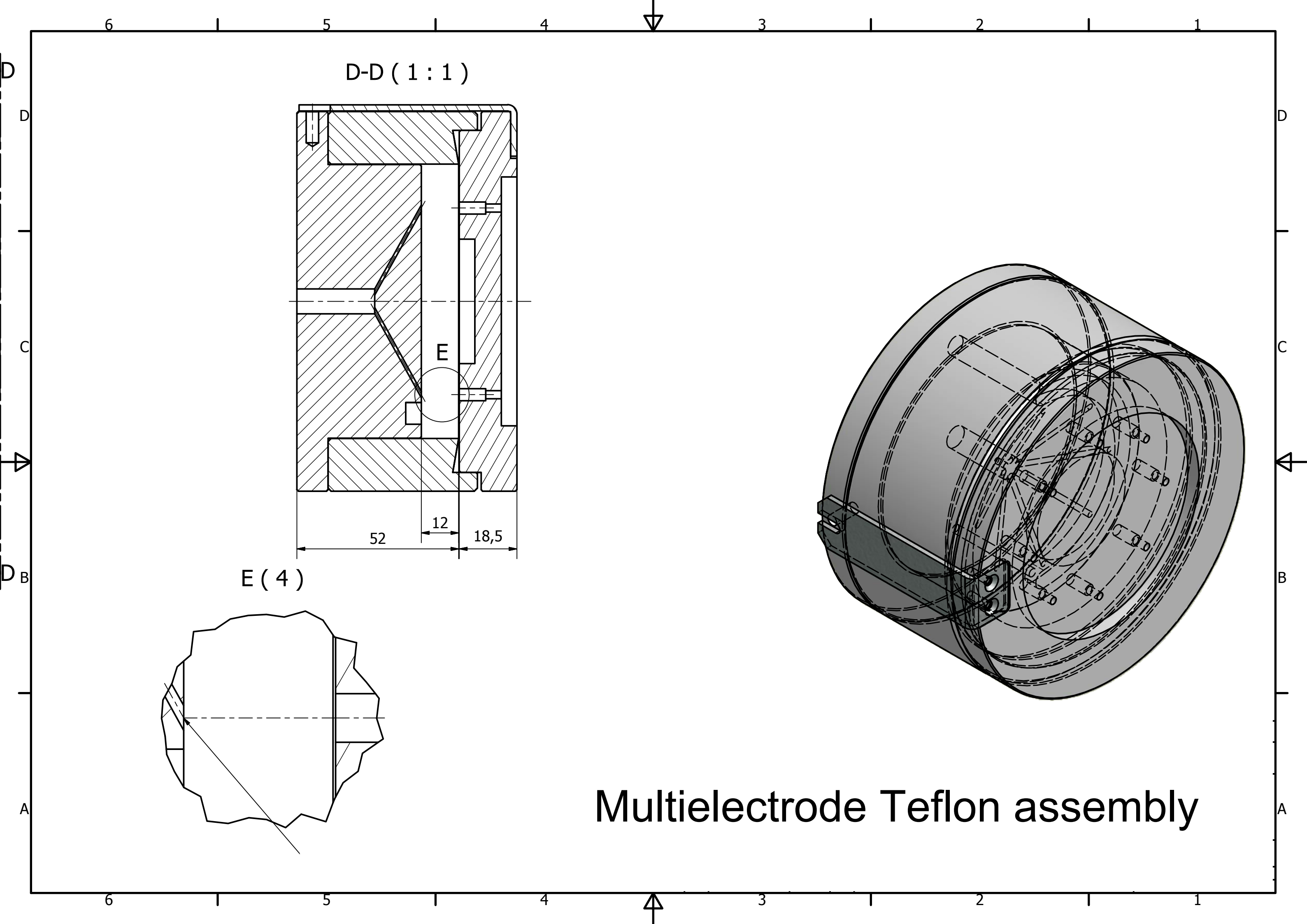

Supplement: Supplementary file 1 [file mmc1.pdf]
